# Modulating effects of mycotoxin and oxidized oil on intestinal microbiota in broiler chickens

**DOI:** 10.1371/journal.pone.0314821

**Published:** 2025-03-03

**Authors:** Kouassi R. Kpodo, Daniel J. Milliken, Philip M. Campos, Monika Proszkowiec‑Weglarz, Merlin D. Lindemann, Sunday A. Adedokun

**Affiliations:** 1 Animal Biosciences and Biotechnology Laboratory, Agricultural Research Service, United States Department of Agriculture, Beltsville, Maryland, United States of America; 2 Department of Animal and Food Sciences, University of Kentucky, Lexington, Kentucky, United States of America; University of Life Sciences in Lublin, POLAND

## Abstract

Climatic change and increased use of alternative sources of feed ingredients could influence poultry production. Mycotoxin and oxidized oil are two contaminations that may occur in chicken feed as a result of climate change and use of alternative feed ingredients, and these factors may have differential and potentially additive effects on birds’ intestinal microbiota. The study objective was to determine the main effects of corn, oil quality, and their interaction on ileal content, ileal scrapings, cecal content, and whole cecum (content and tissue) microbiota in broiler chickens. Broiler chickens were raised for 21 days post-hatch and fed diet made with regular or mycotoxin-contaminated corn (7,959 ppb of deoxynivalenol, 2.1 ppm of aflatoxin, 23,200 ppb of fumonisin, and 1,403 ppb of zearalenone), and regular or oxidized (148 meq/kg) oil. Bacterial genomic DNA was extracted and sequenced targeting the variable (V3-V4) region of the 16S gene. The bioinformatic and statistical analysis of the microbiota data showed mycotoxin and mycotoxin by oxidized oil interaction increased the richness and evenness in the ileal content and only evenness in the cecal content. Mycotoxin and mycotoxin by oxidized oil interaction also increased beta diversity based on the variability in microbial community in the ileal content while increasing the abundance of bacterial taxa, including *Streptomyces* and *Escherichia-Shigella*, and predicted pathways related to RNA and DNA synthesis (Mycothiol and pyrimidine deoxyribonucleotides synthesis) and redox regulation (ergothioneine biosynthesis) in ileal content and pathways related to glycol metabolism and degradation and amino acids degradation were increased in the cecal content. *Streptomyces* has been associated with mycotoxin detoxication, and its increase could reduce the negative effects of mycotoxins contrary to *Escherichia-Shigella*, which has been negatively correlated with weight gain in chickens. These results show that mycotoxin alone and its combination with oxidized oil affect bacterial diversity and abundance mostly in the ileum content and predicted metabolic pathways across intestinal sections.

## Introduction

Oxidative stress is one of the important threats to the gastrointestinal tract health in broiler chickens. The intestinal epithelium is particularly affected by oxidative stress because it is in direct and constant contact with intestinal chyme and potential harmful substances [1], including mycotoxins and oxidized oils. Soybean oil is one of the most used oils as an energy source in poultry diets to meet the energy requirements for modern, fast-growing broiler chickens. It increases the absorption of fat-soluble vitamins, the palatability of the feed, the coefficient of total trac digestibility of fatty acids [2], and decreased abdominal fat [3]. However, soybean oil contains unsaturated fatty acids with one, two, and three double bonds [4,5], which make it more susceptible to lipid peroxidation [6]. Oxidized soybean oil has been shown to decrease the antioxidant capacity in the jejunum [7] and ileum [8], induce inflammation in the small intestine [9], increase lipid peroxidation in the jejunum and the liver of broiler chickens [10], and downregulate tight junction proteins including claudin-1 and Occludin suggesting a disruption in intestinal barrier function [11]. In addition to oxidized oils, other factors such as mycotoxins can contaminate feed ingredients and cause oxidative stress in the intestine of broiler chickens.

Mycotoxins are secondary metabolites naturally produced by fungi that grow on crops and foods, including most cereals (corn, wheat) and other feed ingredients (soybean meal) used in chickens feed. Some of the most prevailing mycotoxins include aflatoxin, fumonisin, zearalenone, and deoxynivalenol. Although these mycotoxins are produced by different fungi such *as Fusarium spp*. (fumonisin, zearalenone, and deoxynivalenol) and *Aspergillus spp*. (aflatoxin), their co-occurrence in cereals is common [12]. Feed can be contaminated in the field or during storage with these mycotoxins, which can negatively affect intestinal functions and overall cellular processes [1,13], resulting in economic losses in the poultry industry [14]. The chronic ingestion of deoxynivalenol and fumonisin reduced body weight gain and nutrient digestibility [15,16], while aflatoxin and zearalenone decreased weight gain and feed conversion ratio in broiler chickens and impaired liver metabolism and functions [17]. The contamination of feed with aflatoxin, deoxynivalenol, and zearalenone decreased weight gain and jejunal villus height and villus height to crypt depth ratio [18]. Nutrient digestion occurs mainly in the intestinal lumen and at the brush border membrane of the enterocytes. The intestinal lumen and the mucosal surface harbor a complex and dynamic microbiota, which plays an important role in intestinal health and digestive functions [19]. Intestinal microbiota have been correlated with greater nutrient utilization by the host [20] and the development and activation of the immune system [21].

Intestinal microbiota is under constant influence of dietary xenobiotics such as toxins and peroxides, and its fluctuations can cause dysbiosis [22,23]. Previous studies have shown that the ingestion of deoxynivalenol [24,25] and fumonisin [26] altered intestinal microbiota, and oxidized soybean oil increased the alpha diversity in the cecum of broiler chickens [11]. However, limited data exist on the combined effects of mycotoxins and oxidized soybean oil on intestinal microbiota and oxidative stress in chickens. In addition, chicken feeds are more likely to be both contaminated with mycotoxins and contain oxidized oils, and their negative effects may be additive on broiler chicken intestine and more precisely on intestinal microbiota. Therefore, the study objective was to determine the effects of mycotoxin-contaminated corn and oxidized soybean oil and their interactive effect on intestinal microbiota in broiler chickens.

## Materials and methods

### Animals, experimental protocol, and tissue samplings

All animal use and care procedures were approved by the University of Kentucky Animal Care and Use Committee (Protocol No: 2022-4157). Day-old male by-product Cobb broiler breeder chickens (n = 256) were obtained from a commercial hatchery at hatch and were assigned to 4 treatments in a factorial arrangement of treatments with 8 birds/cage and 8 replicate cages/treatment in a randomized complete block design with room location as the blocking factor. The corn-soybean meal-based diets were made with either 100% of the regular corn or 100% of the mycotoxin contaminated corn with normal or oxidized soybean oil. The mycotoxin contents of the regular and contaminated corn fines were 531 vs. 7,959 ppb of deoxynivalenol, < 1.3 vs. 2.1 ppm of aflatoxin, 700 vs. 23,200 ppb of fumonisin, and 109 vs. 1,403 ppb of zearalenone. The peroxide values of the regular and oxidized soybean oil were 9.0 and 148 meq/kg, respectively. First, a single basal diet was made without the corn and the soybean oil. Second, the basal diet was divided into the respective experimental diets (equal weight). Third, corn (regular or contaminated) and soybean oil (normal or oxidized) were added to the experimental diets. The experimental treatments were arranged as a 2 x 2 factorial with corn quality (diet with 0% contaminated corn vs. diet with 100% contaminated corn) and oil quality (100% regular soybean oil vs. 100% oxidized soybean oil). Each of the two diets contained 60.5 and 3.5% of the respective corn and oil samples, respectively. Treatment arrangements were mycotoxin (**M**), no mycotoxin (**noM**), normal oil (**nO**), and oxidized oil (**oxO**), resulting in 4 treatment arrangements with mycotoxin and normal oil (**MnO**), mycotoxin and oxidized oil (**MoxO**), no mycotoxin and normal oil (**noMnO**), and no mycotoxin and oxidized oil (**noMoxO**) as their respective interactions. The diet was formulated to meet or exceed the nutrient and energy requirements of broiler (Table 1) as provided by Cobb Nutrition Guide [27], and chickens had unrestricted access to feed and water. They were raised in battery cages (0.61 x 0.51 x 0.36 m) with lighting regimen of 22 h of light and 2 h of darkness until day 21 post-hatch. The room temperature for the first week was set at 32.7°C and was reduced to 30 and 27.2°C, respectively, on days 7 and 14. Throughout the duration of the study (21 days) birds were monitored at least twice daily, between 0700 and 0900 in the morning and 1600 and 1800 in the evening, for any signs of ill health or weight loss up to 15% of the pen average on scheduled weight day were removed and euthanized (usually within an hour of determination). The total mortality was twenty-two birds, twelve of which died naturally and ten of which were removed from the study and humanely euthanized by CO_2_ asphyxiation. Another 32 birds were euthanized for sampling on day 21. Mortalities were not influenced by treatments with 8 (from the positive control treatment), 5, 4, and 5 birds per treatment. On day 21, one bird close to the median weight of the cage was euthanized by CO_2_ asphyxiation prior to sample collection. Ileal content and scrapings were collected from the middle of the ileum, and cecal content and whole cecum (content and tissue) were collected, snap frozen in liquid nitrogen, and stored at −80°C until bacterial DNA extraction.

**Table 1:** Ingredients and nutrients composition of the experimental diets (on as-fed-basis).

Corn	Regular	Contaminated	Regular	Contaminated
Oil	Normal	Normal	Oxidized	Oxidized
*Ingredients, g/kg*				
Corn	604.9	0	604.9	0
Contaminated corn	0	604.9	0	604.9
Soybean meal (47% crude protein)	315	315	315	315
Soybean oil (normal)	35	35	0	0
Soybean oil (oxidized)	0	0	35	35
L-Lysine HCl	3	3	3	3
DL-Methionine	3.6	3.6	3.6	3.6
L-Threonine	1.08	1.08	1.08	1.08
Salt (NaCl)	4	4	4	4
Limestone	9.85	9.85	9.85	9.85
Dicalcium phosphate	16.6	16.6	16.6	16.6
Vitamin-mineral premix^1^	2	2	2	2
Titanium dioxide^2^	5	5	5	5
Total	1,000	1,000	1,000	1,000
Calculated and analyzed (in parenthesis) nutrient and energy values (%)				
Crude protein (nitrogen x 6.25)	20.4 (20.3)	20.4 (20.8)	20.4 (20.0)	20.4 (20.7)
Men^3^ (kcal/kg)	3,133	3,133	3,133	3,133
Calcium	0.90 (0.91)	0.90 (0.97)	0.90 (0.89)	0.90 (0.93)
Phosphorus	0.69 (0.58)	0.69 (0.63)	0.69 (0.60)	0.69 (0.62)
Non-phytate phosphorus	0.45	0.45	0.45	0.45

^1^Provided the following quantities of vitamins and micro minerals per kilogram of complete diet: 32 mg of iron from iron sulfate; 8 mg of copper from copper sulfate,; 51 mg of manganese from manganese oxide; zinc oxide, 60 mg; iodine (EDDI), 1.48 mg; selenium as sodium selenite, 0.24 mg; vitamin A (retinyl acetate), 8,820 IU; vitamin D3 (cholecalciferol), 2,822 IU; vitamin E (dl-α-tocopheryl acetate), 26 IU; vitamin K activity, 0.73 mg; thiamin, 1.76 mg; riboflavin, 6.17 mg; pantothenic acid, 14 mg; niacin, 44 mg; pyridoxine, 4 mg; folic acid, 0.88 mg; biotin, 0.18 mg; vitamin B12, 0.02 mg; choline, 383 mg.

^2^Added to the diets as an indigestible marker for nutrient digestibility and utilization calculations. This was added to the diets to address a second objective of the study.

^3^Metabolizable energy corrected for nitrogen.

### Diet nutrient content determination

Ground diets were analyzed for nitrogen, calcium, and total phosphorus. The nitrogen content was determined by combustion method using a LECO Trumac Nitrogen Analyzer (LECO, St. Joseph, MII; method 990.03) [28]. Prior to the determination of Ca and P levels, the diets were digested using nitric and perchloric acid mixture (method 990.08) [29], and concentrations of Ca and P were determined by inductively coupled plasma-optical emission spectroscopy (Thermo Jarrell Ash, Corporation, Franklin, MA).

### DNA extraction, library preparation, and 16S RNA sequencing

Bacterial DNA was extracted using the DNeasy PowerSoil Kit (Qiagen. Valencia, CA) on a QIAcube instrument (Qiagen) following the manufacturer’s protocol. The DNA concentration and quality were assessed using NanoDrop (ThemoFisher Scientific, Inc. Waltham, MA) and electrophoresis (Bioanalyzer 2100, Agilent Technology, Santa Clara, Ca), respectively. The 16S rRNA library was prepared following the Illumina library preparation workflow using PCR primers targeting the variable V3-V4 region of the 16S gene. The pooled DNA library was diluted to a final concentration of 4 pM and mixed with PhiX (Illumina, Inc., 4 nmol) control (20%v/v) and pair-end 2 x 300 bp sequenced using the Illumina MiSeq platform and a MiSeq Reagent Kit v3 (Illumina, Inc.).

### Statistical analysis

Bioinformatic and statistical analysis of the microbiota data was performed using the Quantitative Insights Into Microbial Ecology software (QIIME 2), version 2023.9 [30]. Reads were denoised using the DADA2 pipeline into amplicon sequence variants (ASVs). Taxonomy was assigned to ASVs using the q2-feature classifier classify-sklearn naïve bayes taxonomy classifier [31] against the SILVA-138-99 reference database [32]. Phylogenetic reconstruction was completed by aligning ASVs with MAFFT and using the FastTree2 program. Alpha diversity metrics were used to estimate diversity within samples by measuring species richness and evenness. The Alpha diversity metrics used are Shannon index (richness and evenness), Observed features (ASVs, richness), Pielou’s Evenness, and Faith’s Phylogenetic Diversity (Faith PD, richness). Beta diversity was assessed as a measure of similarity or distance between treatments using weighted and unweighted UniFrac distances [33]. The nonparametric permutational analysis of variance (PERMANOVA) was used to differentiate the UniFrac distances. Principal coordinate analysis (PCoA) was used to visualize the distances between the different groups. Statistical significance was considered at P ≤ 0.05.

Linear discriminant analysis (LDA) effect size (LEfSe) was used to determine significant differences in bacterial abundance among treatment groups [34] and the taxonomy files were collapsed to genus level. The Phylogenetic Investigation of Communities by Reconstitution of Unobserved States (PICRUSt2) version 2.5.2 software was used to predict the functional composition based on marker gene sequences [35]. The function of the microbiota was determined using phylogeny by reconstruction of states with MetaCyc database [36,37] and STAMP v2.1.3 visualization [38]. Alpha diversity, beta diversity, and taxonomy figures were made using the ggplot2 package in R 4.0.3 [39].

## Results

### Alpha diversity of intestinal microbiota

**Fig 1:**
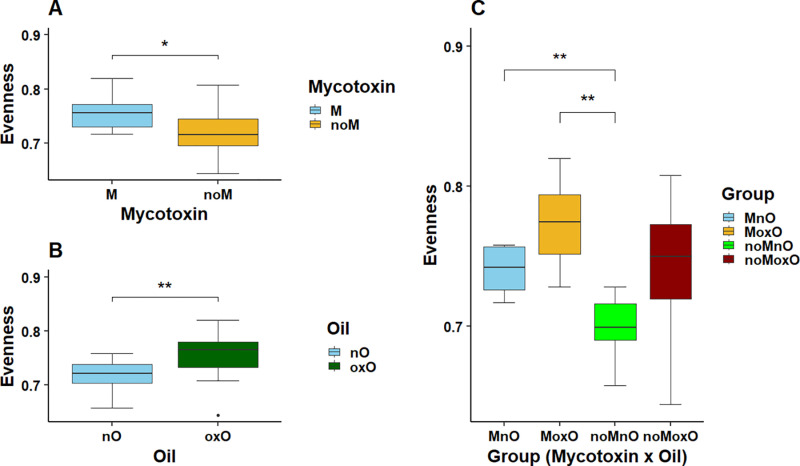
Effects of mycotoxin and oxidized oil on alpha diversity (Evenness) in cecal content bacteria of broiler chickens. Abbreviations are mycotoxin (**M**), no mycotoxin (**noM**), normal oil (**nO**), oxidized oil (**oxO**), mycotoxin and normal oil (**MnO**), mycotoxin and oxidized oil (**MoxO**), no mycotoxin and normal oil (**noMnO**), no mycotoxin and oxidized (**noMoxO**). Asterisks denote statistical significance ( * P < 0.05, ** P < 0.01).

Sequencing summary of ileal scrapings, cecal content, and whole cecum are presented in Table 2. Regarding alpha diversity indexes (ASVs, Shannon, Faith PD, and Evenness), no mycotoxin, oxidized oil effects, or their interaction were observed (P > 0.05) for microbial population in ileal scrapings and whole cecum (Table 3). In cecal content, ASVs, Shannon, and Faith PD for microbial population were not affected (P > 0.05) by M and oxO, or their interaction; however, Evenness was increased by M and oxO (P = 0.019, and 0.007, respectively. Table 3, Fig 1). In addition, Evenness was increased in both MnO and MoxO compared to noMoxO birds (Fig 1).

**Table 2:** Sequencing summary of ileal content, ileal scrapings, cecal content, and whole cecum microbiota dataset processed using qiime2 software. Reads after filtering represent the number of reads after exclusion of mitochondria, chloroplasts, and unassigned bacteria. Abbreviations are amplicon sequence variants (ASVs) and Quality control (QC).

	Ileal content	Ileal scrapings	Cecal content	Whole cecum
Number of samples	28	28	28	28
Raw reads	3,748,859	1,427,341	6,213,415	3,453,566
Reads after QC	1,617,511	20,471,5	2,840,314	934,159
Reads after filtering	1,609,094	204,715	2,840,310	934,159
Reads per sample (range)	10,817–137,054	10–14,578	48,626–254,295	10,380–71,442
Mean reads per sample	57,468	7,311	101,440	33,363
Total number of ASVs	1,475	726	606	412
ASV read length (range)	273–464	290–551	281–501	289–525
Mean ASV read length	431	430	422	426

**Table 3:** Effects of mycotoxin and oxidized oil on alpha diversity indices and beta diversity (PERMANOVA) in ileal scrapings, cecal content, and whole cecum.

		P-values	
Analysis	Ileal scrapings	Cecal content	Whole cecum
Observed ASVs			
Mycotoxin	0.877	0.982	0.421
Oil	0.608	0.089	0.476
Mycotoxin x Oil (Group)	0.522	0.384	0.536
Shannon diversity index			
Mycotoxin	0.918	0.066	0.335
Oil	0.817	0.129	0.613
Mycotoxin x Oil (Group)	0.723	0.124	0.752
Faith’s Phylogenetic diversity			
Mycotoxin	0.681	0.963	0.890
Oil	0.397	0.059	0.854
Mycotoxin x Oil (Group)	0.829	0.301	0.865
Pielou’s Evenneness index			
Mycotoxin	0.643	0.019	0.679
Oil	0.739	0.008	0.927
Mycotoxin x Oil (Group)	0.856	0.005	0.942
PERMANOVA analysis (unweighted UniFrac)			
Mycotoxin	0.985	0.082	0.555
Oil	0.443	0.101	0.911
Mycotoxin x Oil (Group)	0.916	0.075	0.930
PERMANOVA analysis (Weighted UniFrac)			
Mycotoxin	0.945	0.306	0.099
Oil	0.608	0.590	0.469
Mycotoxin x Oil (Group)	0.608	0.442	0.316

Abbreviations are ASVs, amplicon sequence variants; PERMANOVA, permutational multivariate analysis of variance.

**Fig 2:**
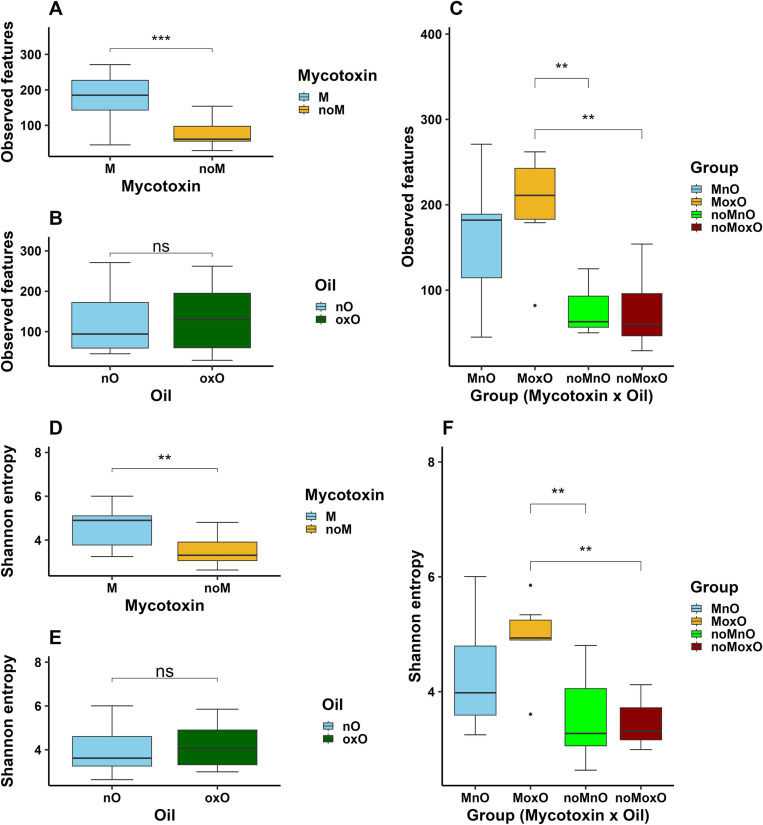
Effects of mycotoxin and oxidized oil on alpha diversity (Observed features and Shannon entropy) in ileal content bacteria of broiler chickens. (**A, D**) mycotoxin, (**B, E**) oil, and (**C, F**) mycotoxin x oil. Abbreviations are mycotoxin (**M**), no mycotoxin (**noM**), normal oil (**nO**), oxidized oil (**oxO**), mycotoxin and normal oil (**MnO**), mycotoxin and oxidized oil (**MoxO**), no mycotoxin and normal oil (**noMnO**), no mycotoxin and oxidized (**noMoxO**), no significance (**ns**). Asterisks denote statistical significance ( *P < 0.05, **P < 0.01).

The most significant differences in alpha diversity were observed in ileal content microbiota. The ASVs were increased by M (P < 0.001, Fig 2A) while no differences were observed between nO and oxO birds (P > 0.05, Fig 2B). There was a M by oxO effect (P < 0.01) where the ASVs were increased in MoxO birds compared to noMnO and noMoxO birds (Fig 2C). Shannon index was increased (P < 0.01) by M (Fig 2D) while no differences were observed (P > 0.05) between nO and oxO birds (Fig 2E). There was a M by oxO effect (P < 0.01) where Shannon index was increased in MoxO birds compared to noMnO and noMoxO birds (Fig 2F).

**Fig 3:**
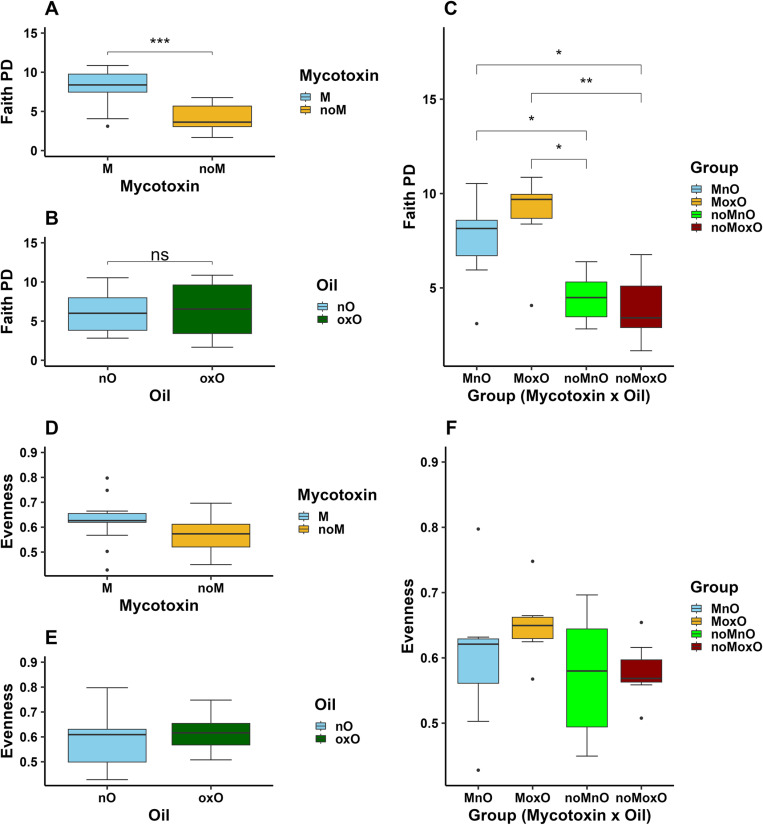
Effects of mycotoxin and oxidized oil on alpha diversity (Faith PD and Evenness) in ileal content bacteria of broiler chickens: (A, D) mycotoxin, (B, E) oil, and (C, F) mycotoxin x oil. Abbreviations are mycotoxin (**M**), no mycotoxin (**noM**), normal oil (**nO**), oxidized oil (**oxO**), mycotoxin and normal oil (**MnO**), mycotoxin and oxidized oil (**MoxO**), no mycotoxin and normal oil (**noMnO**), no mycotoxin and oxidized (**noMoxO**), no significance (**ns**). Asterisks denote statistical significance ( *P < 0.05, **P < 0.01, ***P < 0.001).

Faith PD was increased by M (P < 0.001, Fig 3A) while no differences were observed between nO and oxO birds (Fig 3B). There was a M by oxO effect where Faith PD was increased in MnO birds compared to those fed noMnO and noMoxO diets (P < 0.05), and in MoxO birds compared to noMnO (P < 0.05) and noMoxO (P < 0.01, Fig 3C) birds. No M, oxO, or M by oxO effects were observed (P > 0.05) on Evenness for ileal content bacterial population (Fig 3D–3F).

### Beta diversity of intestinal microbiota

**Fig 4:**
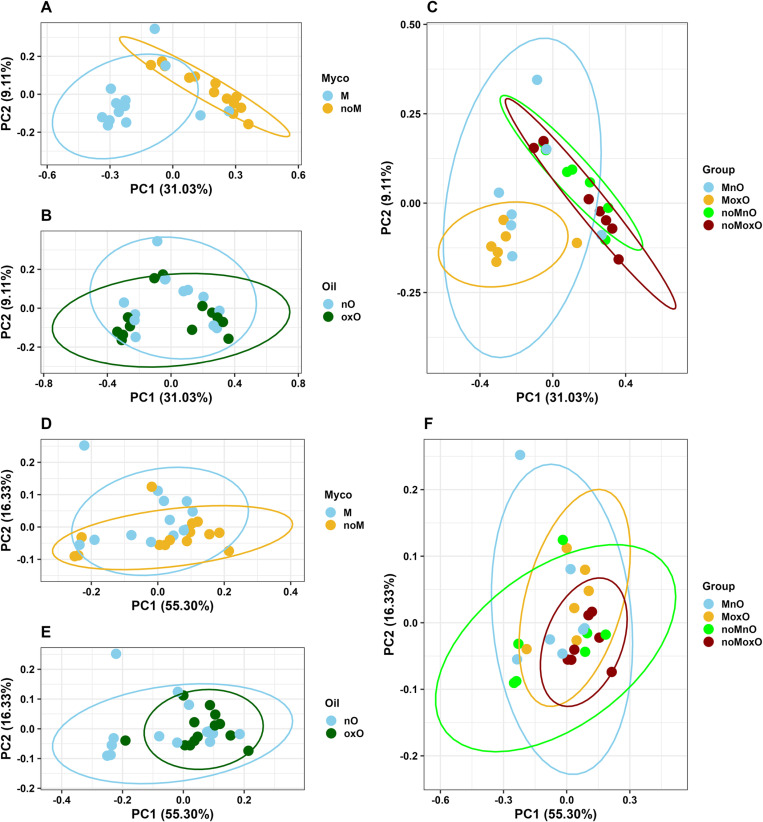
Principal coordinates analysis (PCoA) of ileal content bacteria based on unweighted UniFrac in (A) mycotoxin, (B) oil, and (C) mycotoxin x oil, and on weighted UniFrac in (D) mycotoxin, (E) oil, and (F) mycotoxin x oil. Abbreviations are mycotoxin (**M**), no mycotoxin (**noM**), normal oil (**nO**), oxidized oil (**oxO**), mycotoxin and normal oil (**MnO**), mycotoxin and oxidized oil (**MoxO**), no mycotoxin and normal oil (**noMnO**), no mycotoxin and oxidized (**noMoxO**).

Beta diversity analysis based on unweighted UniFrac revealed significant distinction (P = 0.001) of M from noM birds (Fig 4A) while no distinction was observed between nO and oxO birds (P > 0.05, Fig 4B). There was M by oxO effect for unweighted UniFrac where MnO and MoxO birds were distinct from noMnO and noMoxO birds (P < 0.05, Fig 4C). Beta diversity analysis based on weighted UniFrac revealed no significant differences associated with the presence or absence of M in the diet (P > 0.05, Fig 4D), but there was some separation of nO from oxO birds (P = 0.04, Fig 4E). In addition, no M by oxO effect was observed for weighted UniFrac (P > 0.05, Fig 4F).

### Differential bacterial abundance

**Fig 5:**
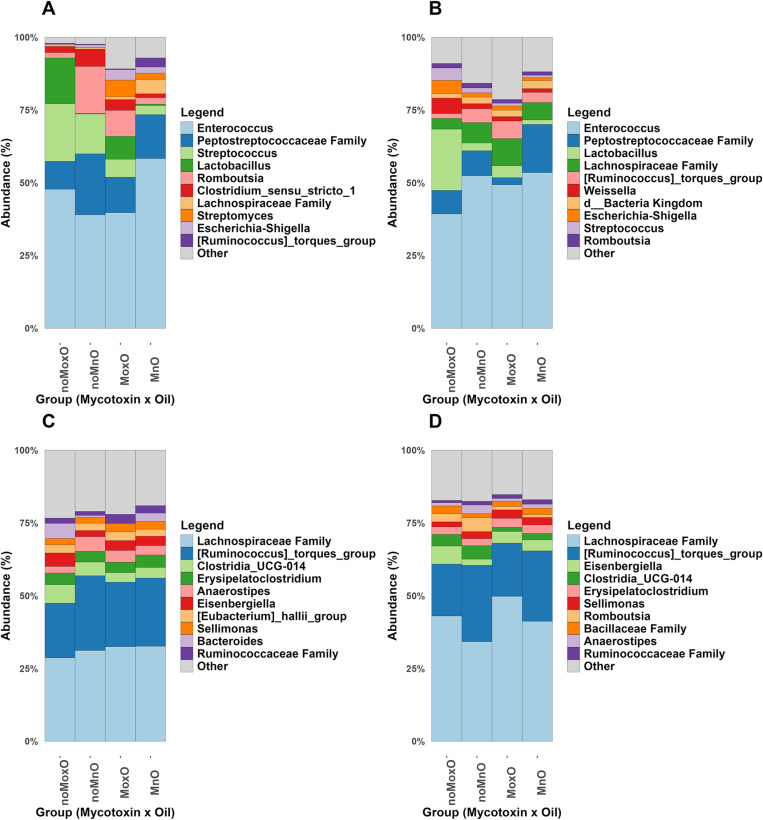
Summary of bacterial composition (relative abundance, %) for mycotoxin and oil interaction at genus level in: (A) ileum content, (B) ileum scrapings, (C) cecal content and (D) whole cecum. Abbreviations are mycotoxin and normal oil (**MnO**), mycotoxin and oxidized oil (**MoxO**), no mycotoxin and normal oil (**noMnO**), no mycotoxin and oxidized oil (**noMoxO**). The 10 most abundant taxa at the genus level are presented in the figure.

In ileal content, the top five taxa were genera *Enterococcus*, *Streptococcus*, *Lactobacillus*, *Romboutsia*, and the family Peptostreptococcaceae. While *Enterococcus*, family Peptostreptococcaceae, and *Streptococcus* are present in all birds, *Lactobacillus* was present in only MoxO, noMoxO and MnO birds (Fig 5A). In ileal scrapings, the top five taxa were genera *Enterococcus*, *Lactobacillus*, and [*Ruminococcus*] *torques group* (brackets indicate contested nomenclature), and the family Peptostreptococcaceae (Fig 5B). In cecal content, the top five taxa were family Lachnospiraceae, [*Ruminococcus*] *torques group*, *Clostridia UCG-014*, *Erysipelatoclostridium*, and *Anaerostipes* (Fig 5C). In whole cecum, the top five taxa were family Lachnospiraceae, [*Ruminococcus*] *torques group*, *Eisenbergiella*, *Clostridia UCG-*014, and *Erysipelatoclostridium* (Fig 5D).

**Fig 6:**
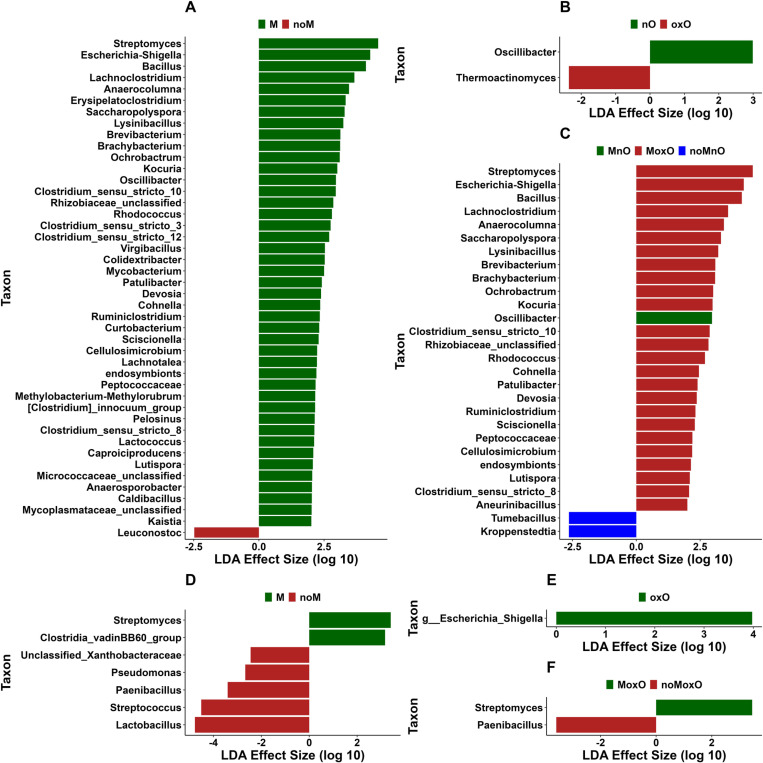
Differential abundance of bacterial population using linear discriminant analysis effect size (LEfSe) of ileal content in (A) mycotoxin, (B) oil, and (C) mycotoxin x oil, and of ileal scrapings in (D) mycotoxin, (E) oil, and (F) mycotoxin x oil. Abbreviations are mycotoxin (**M**), no mycotoxin (**noM**), normal oil (**nO**), oxidized oil (**oxO**), mycotoxin and normal oil (**MnO**), mycotoxin and oxidized oil (**MoxO**), no mycotoxin and normal oil (**noMnO**).

In ileal content, 43 genera were in greater abundance in M birds with the top 5 being *Streptomyces*, *Escherichia-Shigella*, *Lachnoclostridium*, *Anaerocolumna*, and *Erysipelatoclostridium* while only one genus, *Leuconostoc*, was in greater relative abundance in noM birds (Fig 6A). The genera *Oscillibacter* and *Thermoactinomyces* were in greater relative abundance in nO and oxO birds, respectively (Fig 6B). In addition, 25 genera were increased in MoxO with the top 5 being *Streptomyces*, *Escherichia-Shigella*, *Bacillus*, *Lachnoclostridium*, and *Anaerocolumna* while *Tumebacillus* and *Kroppenstedtia* were increased only in noMnO, and *Oscillibacter* was increased in MnO birds (Fig 6C). Two genera (*Tumebacillus* and *Kroppenstedtia*) and the genus (*Oscillibacter*) were in greater relative abundance in MnO and noMnO birds, respectively (Fig 6C).

In ileal scrapings, unclassified Xanthobacteraceae, *Pseudomonas*, *Paenibacillus*, *Streptococcus*, and *Lactobacillus* were increased in noM birds while *Streptomyces* and *Clostridia vadinBB60 group* were increased in M birds (Fig 6D). *Escherichia-Shigella* was increased in oxO compared to nO birds (Fig 6E). In addition, the genera *Streptomyces* and *Paenibacillus* were increased in MoxO and noMoxO, respectively (Fig 6F).

**Fig 7:**
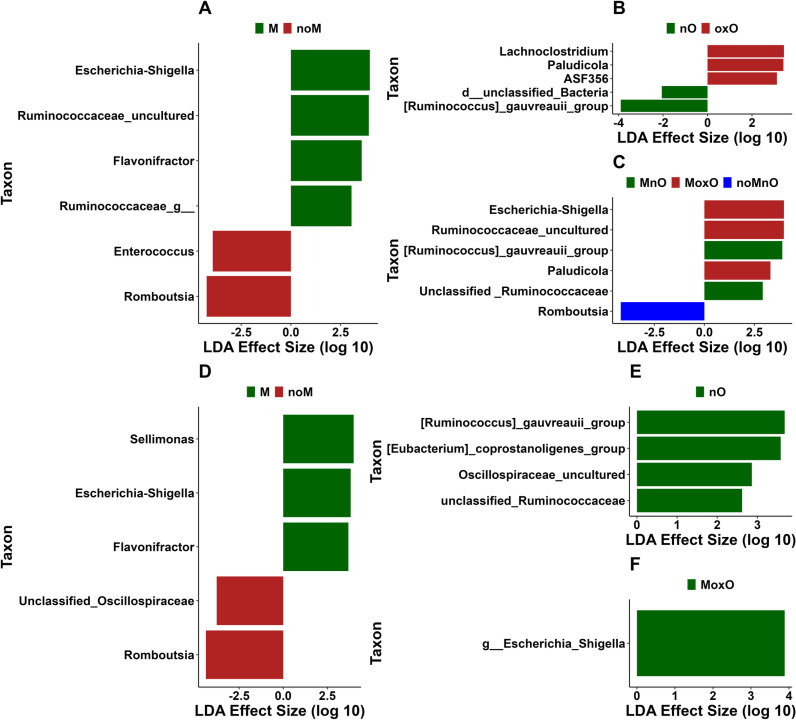
Differential abundance of bacterial population using linear discriminant analysis effect size (LEfSe) of cecal content in (A) mycotoxin, (B) oil, and (C) mycotoxin x oil, and of whole cecum in (D) mycotoxin, (E) oil, and (F) mycotoxin x oil. Abbreviations are mycotoxin (**M**), no mycotoxin (**noM**), normal oil (**nO**), oxidized oil (**oxO**), mycotoxin and normal oil (**MnO**), mycotoxin and oxidized oil (**MoxO**), no mycotoxin and normal oil (**noMnO**).

In cecal content, *Escherichia-Shigella*, uncultured *Ruminococcus, Flavonifractor*, and unclassified Ruminococcaceae were increased in M while *Enterococcus* and *Romboutia* were increased in noM birds (Fig 7A). *Lachnoclostridium*, *Paludicola*, and *ASF356* were increased in oxO while unclassified bacteria, and [*Ruminococcus*] *gauvreauii group* were increased in nO birds (Fig 7B). In addition, *Escherichia-Shigella*, uncultured *Ruminococcus*, and *Paludicola* were increased in MoxO compared to [*Ruminococcus*] *gauvreauii group* in MnO and *Romboutsia* in noMnO birds (Fig 7C).

In whole cecum, *Sellimonas*, *Escherichia-Shigella*, and *Flavonifractor* were increased in M compared to unclassified Oscillospiraceae and *Romboutsia* (Fig 7D). In addition, [*Ruminococcus*] *gauvreauii group,* [*Eubacterium*] *coproscotanoligenes group*, unclassified Oscillospiraceae, and unclassified Ruminococcaceae were increased in nO while no taxa increase where observed in oxO (Fig 7E). The genus *Escherichia-Shigella* was increased in MoxO (Fig 7F).

### Predicted functions of intestinal bacteria

**Fig 8:**
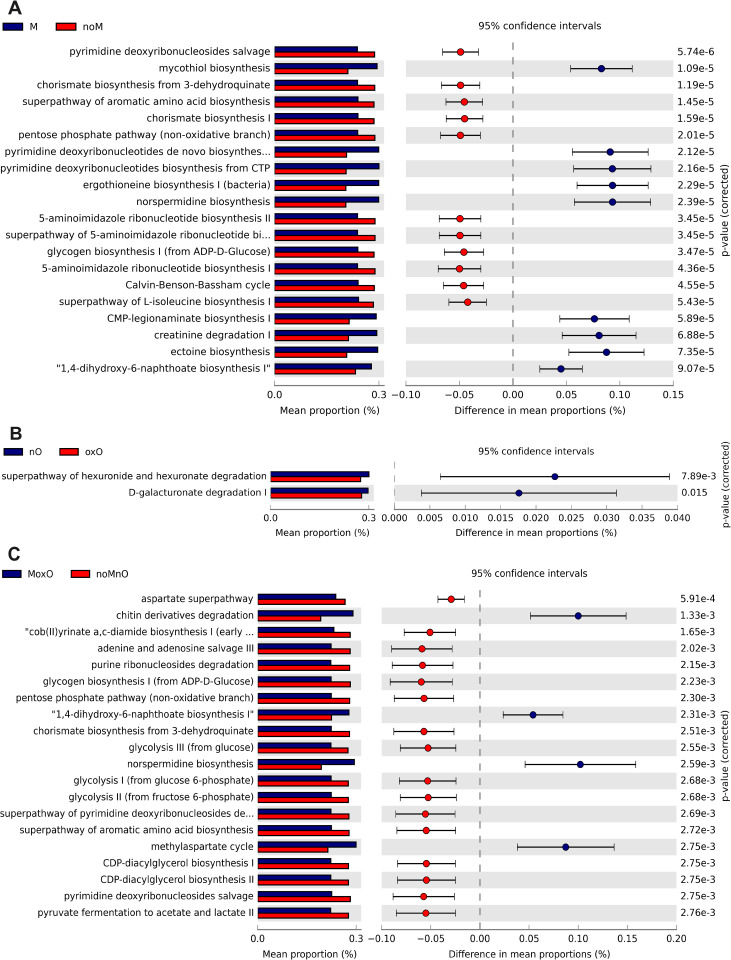
Effects of mycotoxin (A) and oil (B), and (C) MoxO compared to noMnO on predicted functions (only up the top 20 are shown in A and C) of ileal content bacteria. Abbreviations are mycotoxin (**M**), no mycotoxin (**noM**), normal oil (**nO**), oxidized oil (**oxO**), mycotoxin and oxidized oil (**MoxO**), no mycotoxin and normal oil (**noMnO**).

In ileal content, M affected 212 predicted MetaCyc pathways, from which first 20 most significant (P = 0.0001) pathways are shown (Fig 8A). Nine pathways were in greater relative abundance in M birds compared to noM birds (all P < 0.001) including mycothiol biosynthesis, pyrimidine deoxyribonucleotides de novo synthesis, pyrimidine deoxyribonucleotides biosynthesis from CTP, ergothioneine biosynthesis I (Fig 8A). The superpathway hexuronide and hexuronide degradation (P = 0.0008) and D-galacturonide degradation (P = 0.015) were increased in nO compared to oxO birds (Fig 8B). In total, 168 pathways were affected (all P < 0.05) when comparing MoxO to noMnO birds. MoxO increased 49 pathways including chitin derivatives degradation, 1,4-dihydro-6-naphthoate biosynthesis I, norsperimidine biosynthesis, and methylaspartate cycle (top 20 shown in Fig 8C).

In ileal scraping, no predicted pathway differences were observed for M, oxO, and M by oxO interaction (P > 0.05).

**Fig 9:**
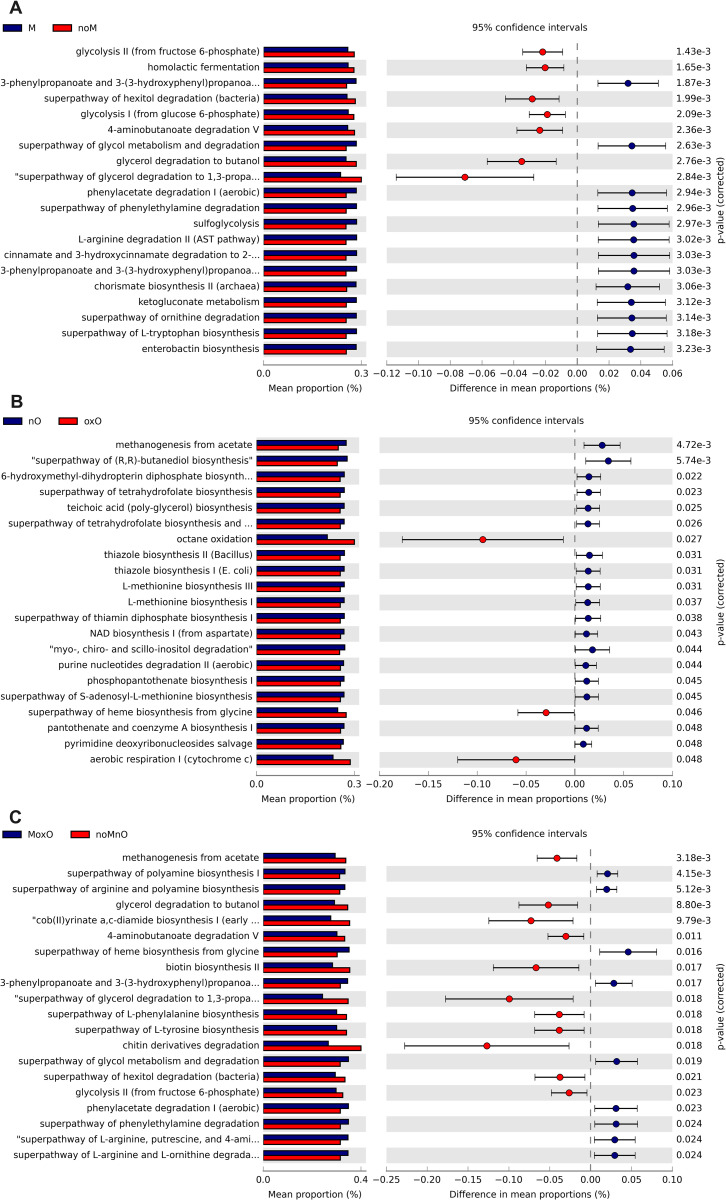
Effects of mycotoxin (A) and oil (B), and (C) MoxO compared to noMnO on predicted functions (only up the top 20 are shown in A, B, and C) of cecal content bacteria. Abbreviations are mycotoxin (**M**), no mycotoxin (**noM**), normal oil (**nO**), oxidized oil (**oxO**), mycotoxin and oxidized oil (**MoxO**), and no mycotoxin and normal oil (**noMnO**).

In cecal content, 41 predicted MetaCyc pathways were increased by M, and the top 20 most significant ones are presented in Fig 9A. Thirteen predicted pathways were in greater relative abundance in M birds compared to noM birds (all P ≤ 0.001), including 3-phenylpropanoate and 3-(3-hydroxyphenyl) propanoate degradation, superpathway of glycol metabolism degradation, phenylacetate degradation I (aerobic), superpathway of phenylethylamine degradation, and sulfoglycolysis (Fig 9A). Twenty-one predicted MetaCyc pathways including octane oxidase superpathway of heme biosynthesis from glycine, and aerobic respiration I (cytochrome c) were greater (all P ≤ 0.001) in oxO compared to nO (Fig 9B). In addition, 40 pathways were affected (all P < 0.05) when comparing MoxO to noMnO birds. MoxO increased 27 pathways including superpathway of polyamine biosynthesis, superpathway of arginine and polyamine biosynthesis, superpathway of heme biosynthesis from glycine, 3-phenylpropanoate and 3-(3-hydroxyphenyl) propanoate degradation, and superpathway of glycol metabolism and degradation, and phenylacetate degradation I (aerobic) (top 20 shown in Fig 9C).

**Fig 10:**
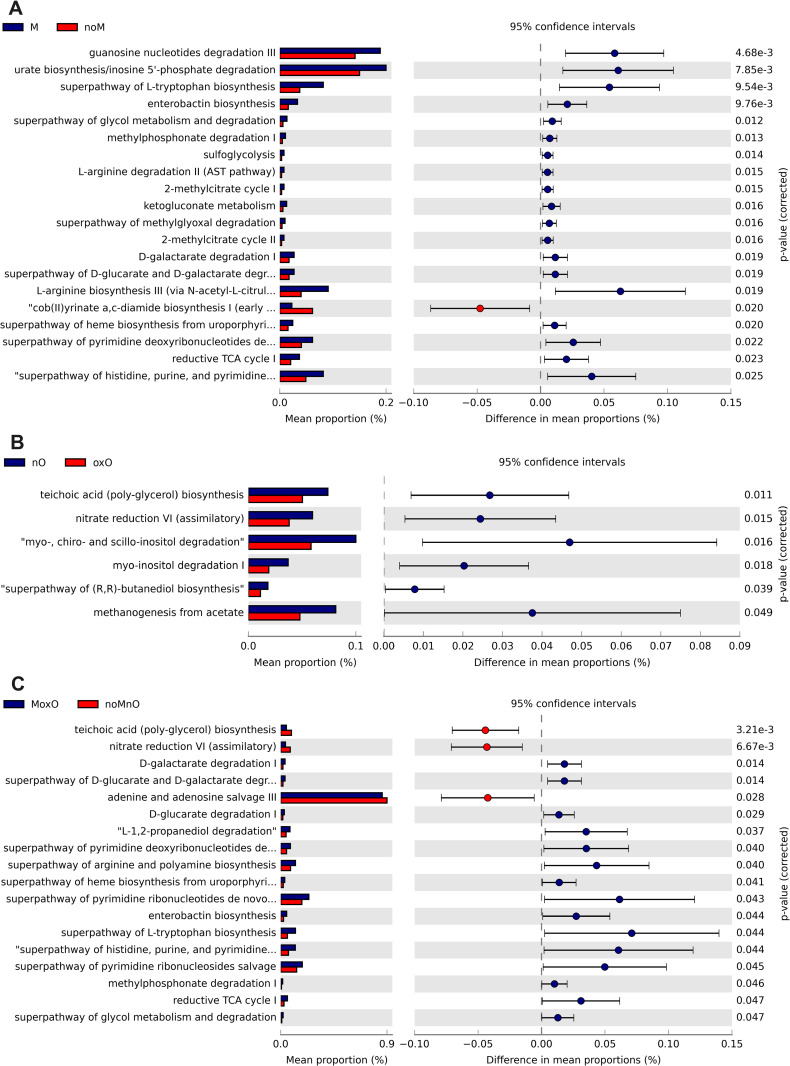
Effects of mycotoxin (A) and oil (B), and (C) MoxO compared to noMnO on predicted functions (only up the top 20 are shown in A and C) of whole cecum bacteria. Abbreviations are mycotoxin (**M**), no mycotoxin (**noM**), normal oil (**nO**), oxidized oil (**oxO**), mycotoxin and oxidized oil (**MoxO**), and no mycotoxin and normal oil (**noMnO**).

In whole cecum, 49 predicted pathways were observed, and the top 20 most significant ones are presented in Fig 10A. Thirteen predicted pathways were in greater relative abundance in M birds compared to noM birds (all P ≤ 0.001) including guanosine nucleotides degradation III, urate biosynthesis/inosine 5’-phosphate degradation, superpathway of L-tryptophane biosynthesis, enterobactin biosynthesis, and superpathway of glycol metabolism and degradation (Fig 10A). Six predicted pathways, teichoic acid (poly-glycol) biosynthesis, nitrate reduction VI (assimilatory), myo-, chiro- and scillo-inositol degradation, myo-inositol degradation I, superpathway of (R,R)-butanediol biosynthesis, and methanogenesis from acetate were in greater relative abundance (all P ≤ 0.05) in nO compared to oxO (Fig 10B). In addition, 18 predicted pathways were affected (all P < 0.05) when comparing MoxO to noMnO birds. MoxO increased 15 predicted pathways including D-galactarate degradation, superpathway of D-glucarate and D-galactarate degradation, D-glucarate degradation, L-1,2-propanediol degradation I, superpathway of pyrimidine deoxyribonucleotides degradation, and superpathway of arginine and polyamine biosynthesis (top 20 shown in Fig 10C).

## Discussion

The adverse effects of mycotoxin and oxidized oil have been associated with intestinal digestive, absorptive and barrier function, but their impacts on intestinal microbiota, which play an important role in intestinal health and functions, have received little attention. Furthermore, most of the published data involved diets with only one or two synthetic mycotoxins; the current study used naturally contaminated corn fines with four different mycotoxins. The interactions of different mycotoxins may result in additive, synergistic, and/or antagonistic effects [40]. Because an average corn grain contains on average 4.8 different mycotoxins [41], this will make predicting mycotoxin interactions from naturally contaminated grain difficult. In this study, we determined the effects of mycotoxin (deoxynivalenol, aflatoxin, fumonisin, and zearalenone) and oxidized oil on intestinal ileal content, ileal scrapings, cecal content and whole cecal (content and tissue) microbiota. Alpha diversity indices (ASVs, Shannon, Faith PD, and Evenness) were used to measure species richness, evenness, or both within a single sample. In the current study, no alpha diversity indices differences were observed in ileal scrapings, whole cecum, and cecal content, except evenness, which was increased in MnO and MoxO birds compared to noMnO birds in the cecal content. Limited data exist on the effects of the combination of all four mycotoxins on intestinal microbiota in chickens. The increase in evenness by M contradicts previous results reported in the cecum of broiler chickens fed deoxynivalenol up to 10mg/kg of feed [24]. The differences could be explained by factors including diet and age of the birds. In the current study, birds were fed diets containing mycotoxin-contaminated corn and samples were collected at 21 days post-hatch while in the previous study [24], diets were contaminated with purified deoxynivalenol, and birds were euthanized from 34 to 37 days post-hatch. Reasons for the lack of M effects on cecal content and tissue microbiota in the current study is unclear; however, the intestinal sections are characterized by different bacterial populations [42], which may impact biological detoxification of deoxynivalenol [43] during its transit and possibly reducing its negative effect on intestinal microbiota in the cecum. In addition, the richness was not affected in the current study contrary to previous data showing an increase in cecal bacterial richness when chicken feed contained aflatoxin [44]. Again, the differences could be because in this study a simple mycotoxin was used compared to the current study. However, the two hypotheses need to be further clarified in future studies.

Oxidized oil also increased evenness in the cecal content indicating some changes in alpha diversity. Contrary to current study, alpha diversity measured through Chao1, and Shannon did not show any differences induced by oxidized soybean oil [8]. Limited data exists on the effects of oxidized oil on cecal microbiota diversity, and more research is needed to better understand the effects of oxidized oil on alpha diversity in the chicken intestine. In addition to the main effects of M and oxO, evenness in cecal content microbiota was increased in MoxO birds compared to MnO and noMnO. The changes in microbiota evenness in the cecal content suggest that feeding mycotoxin and oxidized oil separately or in combination may have caused bacteria species to be more equally distributed in the cecum of the birds. In the ileum content, alpha diversity indices, ASVs, Faith PD, and Shannon, were mostly increased by M and M by oxO interaction. These results agree with a previous report in which a co-occurrence of aflatoxin zearalenone increased jejunal content bacteria richness in broiler chickens [45]. The results of the current study suggest that the presence of M alone or its combination with oxO could increase ileal bacterial diversity although no differences were observed when evenness index alone was considered. Similar to alpha diversity, beta diversity indices were mainly affected only in the ileal content. Beta diversity based on unweighted UniFrac was affected by M and M by oxO interaction. These results show that M and M by oxO interaction have affected the presence or absence of bacterial taxa in the ileal content and less on their abundance since the weighted UniFrac was not affected.

In both the ileal content and ileal scrapings, several genera were present in all the groups (MnO, MoxO, noMnO, and noMoxO). *Enterococcus* is a genus of lactic acid bacteria family and are commensal bacteria in chicken intestine. Although some of *Enterococcus spp.* have been used as probiotics in chickens [46,47], others can cause infection when there is dysbiosis in the intestine [48]. The Peptostreptococcaceae family is a butyric acid producing bacteria commonly found in chickens’ ileum [49]. Contrary to ileal content and scrapings, all the treatment groups in cecal content and whole cecum were dominated by family Lachnospiraceae and [*Ruminococcus*] *torques group*, a genus within Lachnospiraceae. These two taxa are known to degrade cellulose and produce butyrate [42,50]. In addition, *Romboutsia*, which was only present in whole cecum and in all treatment groups, has been associated with the degradation of complex polysaccharides and the production of short chain fatty acids [50].

In addition to the relative abundance, we performed differential abundance analysis to determine taxa that were affected by M, oxO, or M by oxO interaction. The presence of M has increased the abundance of several taxa in ileal content compared to oxO, which affected only one taxon. This result is likely related to the pattern of bacterial presence and absence seen in the alpha and beta diversity results in this study, where observed ASVs were increased in M birds and microbiota were distinct between M and noM birds in ileal content. Together, these results suggest that M results in the presence of bacteria that would typically be absent from the microbiota, leading to distinct microbiota between M and noM birds. Overall, feed contamination by M may have a bigger effect on the bacterial community compared to oxO. However, more research is needed to better understand the effects of M and oxO on intestinal microbiota in broiler chickens. Considering the interaction effects, MoxO induced more differentially abundant bacteria confirming again that the presence these two contaminants, M and oxO, may not only increase the richness as previously indicated but also the abundance of bacteria that were present in the intestine. *Streptococcus* relative abundance was increased in the ileal content of noM birds compared to M birds. The function of *Streptococcus* species can vary, with some strains isolated from the chicken cecum having positive associations such as butyrate production [51] or probiotic potential [52], while in other cases *Streptococcus* can be pathogenic and has been negatively correlated with body weight gain in poultry [53]. As we would expect diets with M to reduce body weight gain, we hypothesize the *Streptococcus* in our study could be positively correlated with weight gain, however, further study to identify their function is required. *Lactobacillus* relative abundance was also significantly greater in noM birds in the ileal content, which appears to be due to higher relative abundance in the noMoxO group, as the relative abundance in noMnO was lower in comparison. This may indicate that the particular combination of noM and oxO could provide conditions beneficial to *Lactobacillus* in terms of relative abundance. *Lactobacillus* is generally the primary bacteria in chicken ileum and reasons why this genus was increased in this treatment groups are unclear.

The most abundant taxa across all segments of the intestine are *Streptomyces*, *Escherichia-Shigella*, and *Bacillus*. The genus *Streptomyces* generally lives in the environment [54]; however, it has been detected in the chicken cecum of free range laying compared to caged laying hens. Some species of genera *Streptomyces* and *Bacillus* have been associated with M detoxification including aflatoxin and zearalenone [55], and deoxynivalenol, respectively [43,54], and this could explain why these two genera were increased in mycotoxin-fed birds in the current study. Another genus, *Escherichia-Shigella*, found in relatively high abundance in the intestine of broilers in the current study, was related to the presence of M and oxO. *Escherichia-Shigella* is generally found in the intestine of chickens; however, its abundance in M and oxO birds may reduce production performance since the abundance of *Escherichia-Shigella* has been negatively correlated with nutrient digestion and weight gain [56].

The relative abundance changes observed in the presence of M and oxO may have affected intestinal bacterial metabolism based on prediction of metabolic pathway abundances. It should be considered that accuracy of functional prediction can be limited outside of human studies [57], however, the insight may inform future studies to better understand functioning within the microbiota. Several metabolic pathways have been predicted to increase in the current study, including mycothiol, pyrimidine deoxyribonucleotides, ergothioneine, norsperimidine synthesis in the ileum and the cecum. Mycothiol is a small thiol, analogous to glutathione, produced by *Actinobacteria* [58] that plays an important role in redox regulation. In addition, ergothioneine biosynthesis I is known to be involved in redox regulation and bacterial survival in stressful conditions [59]. The increase in mycothiol and ergothioneine synthesis may be an adaptation to the M environment because mycotoxins cause oxidative stress in the intestine [18,26,60]. Two other pathways, pyrimidine deoxyribonucleotides de novo synthesis and pyrimidine deoxyribonucleotides biosynthesis from CTP, were increased in M and MoxO birds. The increase in bacterial relative abundance in M and MoxO birds suggests that more RNA and DNA would be synthesized to support cell multiplication and growth, and this could explain the increase in these pathways. Norsperimidine is a polyamide that is associated with high growth rate and active cell division in bacteria [61]. It is also involved in the formation of biofilm, which allows bacteria to colonize their habitat [62], and the increase in norsperimidine pathway may be necessary for the abundant bacteria to effectively occupied their niche in the ileal lumen.

In the cecal content, the predicted genes associated with the presence of M and MoxO are related to degradation pathways including superpathway of glycol metabolism and degradation, amino acids such as L-arginine, L-ornithine, and phenylalanine degradation. All these pathways were increased in M and MoxO birds. Generally, proteins and amino acids that bypass digestion in the small intestine are subjected to fermentation and degradation by bacteria in the cecum [63]. The increase in amino acids degradation pathways in M and MoxO suggests that more proteins of the diet could have escape digestion in the small intestine and the amino acids degradation pathways were increased as a result.

## Conclusion

Mycotoxins and oxO are two major contaminants that negatively affect intestinal function and possibly the microbiota in chicken intestine. In this study, we determined the effects of M, oxO, and M by oxO interaction on intestinal microbiota. The results showed that M and M by oxO interaction affected bacterial richness and evenness mostly in the ileal content while evenness was the only alpha diversity index affected in cecal content. Bacterial differential abundance and predicted functions suggested that M, oxO, and MoxO changed metabolic pathways in the intestine of the birds. Although oxO had limited effects on alpha and beta diversity, it reduced some predicted bacterial functions in the intestine, and more research should be conducted on the effects of oxO alone on intestinal microbiota and functions in broiler chickens. Because the contamination of chicken feed with M and oxO will likely occur in commercial setting, more research should be conducted to develop mitigation strategies to reduce the negative impacts of M and oxO additive effects on intestinal bacterial profile and functions.
